# Non-Contact Transmission Line Galloping Detection Method Utilizing Frequency and Phase Features of Tower-Side Multi-Measuring-Point Magnetic Field

**DOI:** 10.3390/s26133973

**Published:** 2026-06-23

**Authors:** Jun Chen, Jie Wu, Libing Tao, Luheng Huang, Zhuoru Ye, Yalong Mai

**Affiliations:** 1 Quzhou Power Supply Company, State Grid Zhejiang Electric Power Co., Ltd., Quzhou 324000, China; chenjun2006619@163.com (J.C.); 13505708332@163.com (L.T.); 15138000569@139.com (L.H.); 15808553893@163.com (Z.Y.); 2 Yangtze River Delta Research Institute (Quzhou), University of Electronic Science and Technology of China, Quzhou 324004, China; jiew@uestc.edu.cn; 3 School of Mechanical and Electrical Engineering, University of Electronic Science and Technology of China, Chengdu 611731, China

**Keywords:** transmission line, conductor galloping, magnetic sensing, sensor array

## Abstract

Non-contact magnetic sensing technology is widely adopted in transmission line online monitoring scenarios including current measurement and fault location for its non-contact measurement capability, strong environmental robustness and low deployment cost. However, existing magnetic-sensing-based galloping monitoring methods suffer from two critical limitations: no theoretical guidance is provided for sensor placement, and a high false detection rate is observed under current fluctuation conditions. To address these issues, a novel transmission line galloping monitoring method based on spatial magnetic field distribution features is proposed in this paper. A conductor galloping-power frequency magnetic field coupling model is first established to derive the optimal magnetic sensor array arrangement strategy. Subsequently, a galloping detection algorithm fusing multi-node frequency-domain features and phase difference information is proposed to eliminate current fluctuation induced false detection. Simulations conducted based on actual 500 kV transmission line parameters and verification tests carried out on a scaled-down laboratory platform confirm that reliable galloping detection can be realized by the proposed method under both current low-frequency oscillation and random fluctuation scenarios. With advantages of non-contact deployment, high anti-interference performance and detection accuracy, the proposed method has promising application potential in engineering-oriented high-voltage transmission line monitoring.

## 1. Introduction

Transmission lines serve as the core transmission carriers of the modern Energy Internet, undertaking the critical missions of cross-regional power transmission and regional power supply stability maintenance. Their safe operation is directly associated with the reliability of power systems and the order of national economic production [1,2,3,4,5]. Most transmission lines are erected in open wild areas and exposed to complex natural environments for a long period, making them vulnerable to extreme meteorological disasters including strong winds, ice accretion, and lightning strikes [6,7,8]. Among all types of line faults, conductor galloping (CG) induced by iced conductors under steady laminar wind is one of the most hazardous events: as a low-frequency large-amplitude oscillation phenomenon with a frequency range of 0.1–3 Hz and amplitude up to 20–300 times the conductor diameter, galloping may cause mild consequences such as line flashover, tripping, and power fitting wear in minor cases, and even trigger severe accidents including conductor breakage and tower collapse in extreme scenarios [9,10,11,12,13]. Accordingly, high-precision and highly reliable real-time monitoring of transmission line galloping conditions is widely acknowledged as the core prerequisite for implementing galloping early warning, active intervention, and guaranteeing the safe and stable operation of power grids [14,15,16,17].

The current mainstream transmission line galloping monitoring technologies are mainly divided into three categories: image vision-based methods, inertial measurement unit (IMU)-based methods, and fiber Bragg grating (FBG) sensing-based methods. For image vision-based monitoring technologies, core parameters including galloping amplitude and frequency are inversed from collected spatial shape images of conductors, relying on computer vision and image feature extraction technologies: ref. [18] proposes a non-contact monitoring method for transmission line galloping based on infrared source target detection. By deploying infrared reflective tags and combining an image tracking algorithm, it can be adapted to complex scenarios such as nighttime, rain and fog, with deployment costs far lower than those of traditional contact-based solutions. In [19], researchers propose a video monitoring scheme for the galloping of catenary additional conductors in the strong wind zone of the Lanzhou–Xinjiang high-speed railway. Adopting video surveillance and image optimization processing algorithms, it realizes accurate extraction and real-time online monitoring of conductor galloping parameters. Researchers in [20] propose a simple marker-free analysis method for transmission line galloping based on video monitoring and Tracker software. Taking the tower height as a fixed scale, it tracks the trajectory of the lowest point of the conductor sag frame by frame, and can extract galloping frequency and amplitude without additional features such as spacer bars. However, such methods have poor environmental robustness: feature extraction fails under low-visibility/low-illumination scenarios such as rain, fog, snow, and night, leading to a sharp drop in recognition accuracy. High edge-side computing power deployment and equipment operation and maintenance costs result in insufficient economic efficiency for large-scale promotion. For IMU-based monitoring technologies, inertial measurement units integrated with accelerometers and gyroscopes are mounted on the conductor surface to directly collect raw data such as motion acceleration and angular velocity of conductors, and full galloping parameters are obtained through attitude calculation: ref. [21] proposes a wireless remote monitoring scheme for wind-induced galloping of transmission lines. It adopts a self-powered MEMS sensor array and the Orthogonal Matching Pursuit (OMP) algorithm, realizing the distortion-free reconstruction and accurate monitoring of long-span galloping curves. Scholars in [22] propose a wireless monitoring method for wind-induced vibration of high-voltage transmission lines. A wireless sensing system is constructed using MEMS accelerometers and energy harvesting technology, which realizes the measurement of conductor fatigue indexes and the accurate identification of wind-induced vibration types. Ref. [23] proposes a wireless monitoring sensor module for transmission line galloping based on an inertial measurement unit (IMU). It unifies the coordinate reference through quaternion transformation, and reconstructs the three-dimensional galloping trajectory combined with Bezier curve fitting, eliminating the measurement error caused by conductor torsion. However, terminals of such technologies need to be mounted on high-voltage energized conductors, so installation and operation and maintenance require line power outage operations, with high operation thresholds and great implementation difficulty. In addition, inertial sensors have inherent cumulative drift errors, so the measurement accuracy of continuous operation decays continuously with operating time. For FBG-based monitoring technologies, relying on the strain/temperature sensitivity characteristics of fiber Bragg gratings, FBG sensing strings are compounded inside overhead ground wires or OPGW optical cables. The strain change of conductors is inversed by demodulating the offset of the grating center wavelength, and finally the galloping state parameters are obtained: Researchers [24] propose an FBG (Fiber Bragg Grating) fitting-type monitoring method for transmission line tension and galloping. It adopts three-dimensional force detection and temperature compensation technology, realizing all-weather accurate real-time monitoring of tension, galloping modes and amplitudes. Yan, Q et al. [25] propose a distributed optical fiber monitoring scheme based on chirped FBG array enhanced Φ-OTDR (Phase-sensitive Optical Time Domain Reflectometer). Using drawing tower grating array sensing technology, it realizes the accurate and reliable detection of transmission line galloping shape, frequency and amplitude. An FBG dynamic tension monitoring scheme for transmission line galloping is proposed in [26]. It adopts an S-shaped elastic structure and differential temperature compensation technology, realizing passive remote, high-precision and stable detection of galloping parameters. The common disadvantage of such methods is an extremely high deployment threshold: application on existing lines requires replacement of original optical cables, so they are only applicable to new-built line scenarios. Additionally, the temperature cross-sensitivity problem cannot be completely eliminated, leading to large measurement errors and insufficient accuracy stability under wide temperature fluctuations of −20–60 °C in the field. In summary, the three types of existing mainstream technologies all have common shortcomings such as limited applicable scenarios, insufficient long-term reliability, and excessive deployment and operation and maintenance costs. Therefore, a new galloping monitoring technology with no requirement for conductor contact, no interference from environmental factors, and controllable costs is urgently needed to fill the current technical application gap.

In recent years, non-contact sensing technology based on spatial magnetic field (MF) inversion has been proposed as a novel technical solution for transmission line galloping monitoring [27,28,29,30,31,32,33]. During the operation of AC transmission lines, a stable power-frequency magnetic field is generated in the surrounding space, and its spatial distribution characteristics have a strict corresponding relationship with the spatial position of conductors. By deploying a low-power magnetic sensing array on the tower side, parameters including galloping amplitude, frequency and mode of conductors can be accurately obtained through the inversion of magnetic field distribution changes. This technology requires no contact with high-voltage energized conductors, is not interfered by environmental factors such as illumination, temperature and visibility, and requires no modification of existing line structure for device deployment. With multiple advantages of high measurement accuracy, strong long-term operation reliability and low difficulty in large-scale promotion, it provides a feasible direction for the iterative upgrading of galloping monitoring technology. As can be seen from Table 1, the magnetic field sensing method offers comprehensive advantages in non-contact installation, low cost, all-weather adaptability, low maintenance, and minimal damage risk. It overcomes the vulnerabilities of contact-type sensors and the severe weather dependence of vision-based approaches, demonstrating promising potential for online galloping monitoring of transmission lines.

However, existing magnetic sensing-based galloping monitoring technologies are still immature, and no effective solutions have been proposed for the following pending problems in the field:

1. The deployment of sensors on towers lacks reliable theoretical support, making it impossible to be applied in actual engineering scenarios.
2. Magnetic field changes caused by current fluctuations or faults will lead to inaccuracy of magnetic field-based galloping identification methods.

To address the above gaps, a transmission line galloping monitoring method based on spatial magnetic field distribution characteristics is proposed in this paper. First, the sensitivity difference of spatial positions to different galloping modes (in-plane and out-of-plane galloping) is explored based on the galloping-magnetic field distribution model, and the optimal arrangement strategy of magnetic sensor arrays for galloping monitoring is determined, which provides theoretical support for sensor installation in actual scenarios. Second, to solve the problem of false detection in galloping identification caused by current fluctuations, the frequency-domain characteristics of magnetic fields under current fluctuation and normal galloping scenarios are analyzed, and a galloping detection method combining frequency-domain and phase information is proposed, which is not affected by current fluctuations. The effectiveness of the method is verified through simulation experiments with actual line parameters, and its feasibility is further verified on a scaled experimental platform built in the laboratory. The main innovations of this paper are as follows:

1. A spatial magnetic field distribution characteristic model under galloping conditions is established, and a magnetic sensor array arrangement method for galloping detection is proposed on this basis, which can detect the galloping of different modes and has current interference resistance capability.
2. A galloping detection algorithm based on magnetic field frequency-domain and phase analysis is proposed, which effectively eliminates the influence of magnetic field interference caused by current fluctuations on magnetic field-based galloping detection.
3. The feasibility of the proposed galloping detection method is verified through simulations based on actual tower parameters, and a scaled-down laboratory model is built to further verify the effectiveness of the method.

## 2. Spatial MF Distribution Model Considering CG

### 2.1. Transmission Line Galloping Modeling

**Figure 1:**
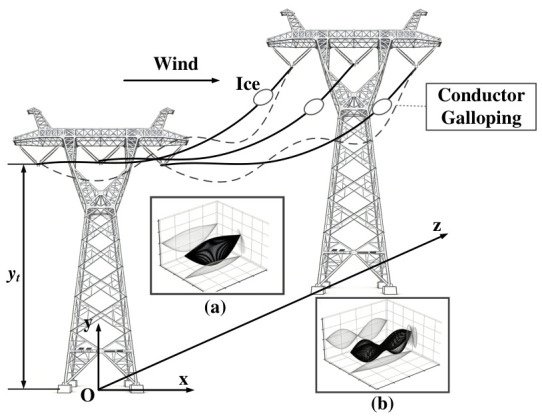
Schematic diagram of typical 500kV transmission line iced galloping: (**a**) first-order mode galloping (**b**) second-order mode galloping.

Research on galloping morphology has evolved from Den Hartog’s transverse vibration theory [34] and Nigol’s torsional vibration theory [35] to the currently widely accepted three-degree-of-freedom galloping theory. In practical scenarios, transverse vibration of galloping is the most extensively studied type, as it exhibits the most significant spatial morphological performance and leads to more severe impacts compared with torsional vibration. Torsional vibration is generally converted into large-amplitude transverse vibration in most cases. Therefore, to simplify the analysis, only the transverse vibration process of galloping is investigated in this paper. When conductor galloping occurs, standing waves with one or multiple antinodes are formed within the span. Figure 1 illustrates the motion trajectory of conductors during galloping, where Figure 1a and Figure 1b present conductor galloping with different morphologies respectively.

Accordingly, the morphology of galloping conductors can usually be described by the following standing wave equation (taking the coordinate axes shown in Figure 1 as the reference):

(1) $$\left\{\begin{array}{cc} y_{p} & =A_{i}sin\left(2\pi f_{i}t+\varphi_{i}\right)sin\left(\frac{n_{i}\pi z_{p}}{L}\right)+y_{st},\\ x_{p} & =A_{o}sin\left(2\pi f_{o}t+\varphi_{o}\right)sin\left(\frac{n_{o}\pi z_{p}}{L}\right)+l_{p}, \end{array}\right.$$

where $A_{i}$ and $A_{o}$ denote the galloping amplitudes of in-plane (vertical direction) and out-of-plane (horizontal direction) modes respectively, $f_{i}$ and $f_{o}$ denote the galloping frequencies, $\varphi_{i}$ and $\varphi_{o}$ denote the initial oscillation phases of galloping, and $n_{i}$ and $n_{o}$ denote the galloping orders, i.e., the number of antinodes formed within the span (the value of *n* is usually no more than 3 in practical scenarios). *L* is the span length, and $l_{p}$ is the phase-to-phase distance. $y_{st}$ represents the conductor profile equation under static state. Affected by gravity, the naturally sagging conductor follows a catenary equation. However, due to the high complexity of the catenary equation, the static profile of conductors can usually be approximated by the parabolic equation [28]:

(2) $$y_{st}=s{\left(\frac{2z_{p}-L}{L}\right)}^{2}+y_{t}-s$$

### 2.2. Modeling of MF Under Galloping Conditions

In accordance with the Biot–Savart Law and existing relevant studies [31], a mathematical model for spatial magnetic field distribution around a single conductor is established:

(3) $$\vec{B}\left(t\right)={\vec{i}}_{x}A_{Xps}\left(t\right)I_{p}\left(t\right)+{\vec{i}}_{y}A_{Yps}\left(t\right)I_{p}\left(t\right)+{\vec{i}}_{z}A_{Zps}\left(t\right)I_{p}\left(t\right),$$

where

(4) $$\begin{array}{cc} \; & A_{Xps}\left(t\right)=\frac{\mu_{0}}{4\pi }\int_{-L}^{L}\frac{\frac{\partial y_{p}}{\partial z_{p}}\left(z_{s}-z_{p}\right)-\left(y_{s}-y_{p}\right)}{{\left(\sqrt{{\left(x_{s}-x_{p}\right)}^{2}+{\left(y_{s}-y_{p}\right)}^{2}+{\left(z_{s}-z_{p}\right)}^{2}}\right)}^{3}}dz_{p},\\ \; & A_{Yps}\left(t\right)=\frac{\mu_{0}}{4\pi }\int_{-L}^{L}\frac{\left(x_{s}-x_{p}\right)-\frac{\partial x_{p}}{\partial z_{p}}\left(z_{s}-z_{p}\right)}{{\left(\sqrt{{\left(x_{s}-x_{p}\right)}^{2}+{\left(y_{s}-y_{p}\right)}^{2}+{\left(z_{s}-z_{p}\right)}^{2}}\right)}^{3}}dz_{p},\\ \; & A_{Zps}\left(t\right)=\frac{\mu_{0}}{4\pi }\int_{-L}^{L}\frac{\frac{\partial x_{p}}{\partial z_{p}}\left(y_{s}-y_{p}\right)-\frac{\partial y_{p}}{\partial z_{p}}\left(x_{s}-x_{p}\right)}{{\left(\sqrt{{\left(x_{s}-x_{p}\right)}^{2}+{\left(y_{s}-y_{p}\right)}^{2}+{\left(z_{s}-z_{p}\right)}^{2}}\right)}^{3}}dz_{p}. \end{array}$$

Here $\mu_{0}$ is the vacuum permeability, $\left(x_{s},y_{s},z_{s}\right)$ denote the position coordinates of an arbitrary point in space, $I_{p}$ is the phase current. Since the magnetic field component along the x-axis has a large magnitude and is highly sensitive to variations of conductor galloping, only the x-axis magnetic field component is investigated in this paper. For a system consisting of *n* conductors, the MF density at a target point can be expressed as

(5) $$B_{Xs}=\sum_{p=1}^{n}A_{Xps}\left(t\right)I_{p}\left(t\right),$$

where $A_{xps}$ is the integral term in (4), whose value depends on the relative position of the sensor with respect to the conductor. However, it is usually not suitable for direct calculation given the complex integral term involved. In the subsequent section, the spatial magnetic field fluctuation characteristics under conductor galloping scenarios for sensors deployed at different positions are analyzed, and the simplified expression of $A_{Xps}$ under corresponding working conditions is derived.

### 2.3. Analysis of MF Distribution Characteristics Under Galloping

Investigation on the spatial magnetic field distribution characteristics during conductor galloping facilitates the identification of positions with the highest sensitivity to galloping, which provides theoretical support for the deployment of magnetic field sensors. According to the above analysis, the relative position between the conductor and any arbitrary point in space changes during galloping, and the magnetic field fluctuation is characterized by the parameter $A_{Xps}$. Therefore, it is necessary to study the expression of $A_{Xps}$ under galloping conditions. Owing to the spatial symmetry of three-phase conductors, the analysis is simplified by taking the variation of $A_{X1s}$ during the galloping of Phase A conductor as a typical case in this paper. The analysis process for galloping of other phase conductors is consistent with the case introduced here.

**Figure 2:**
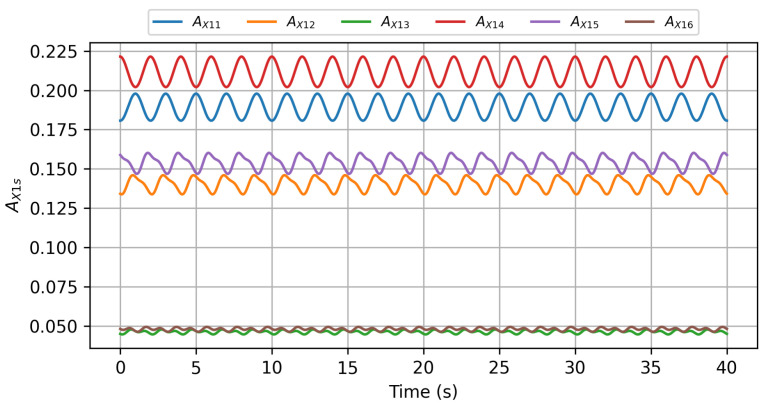
Variation of MF intensity coefficient $A_{X1s}$ at six test points under conductor galloping.

**Figure 3:**
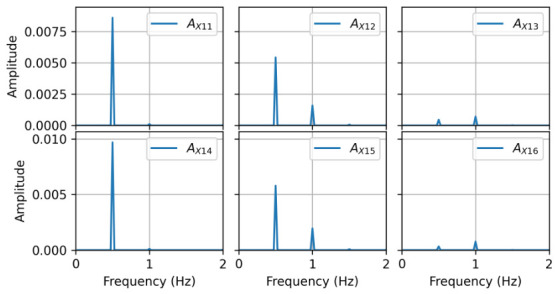
Frequency spectrum of MF intensity coefficient $A_{X1s}$.

Six MF sensors are deployed at six positions to investigate the response of magnetic fields at different locations to Phase A galloping: the horizontal distances from the Phase A conductor are 0 m, 1/3 phase-to-phase distance and 2/3 phase-to-phase distance respectively, with two vertical positions set for each horizontal point, 5 m above the conductor and 5 m below the conductor. In addition, to analyze the influence intensity of in-plane and out-of-plane galloping on the magnetic field at different spatial positions, the in-plane galloping is set with a frequency of 0.5 Hz and an amplitude of 6 m, while the out-of-plane galloping is set with a frequency of 1 Hz and an amplitude of 3 m. With time taken as the independent variable, calculation is carried out at a time interval of 2 ms, and the variation of $A_{X1s}$ within 40 s is calculated. The time-domain and frequency-domain results of $A_{X1s}$ are shown in Figure 2 and Figure 3, respectively.

It can be clearly observed from Figure 2 and Figure 3 that the fluctuations of $A_{X13}$ and $A_{X16}$ at measuring points $S_{3}$ and $S_{6}$, which are far from the Phase A conductor, are negligible during Phase A galloping. They contain almost no frequency components related to Phase A galloping compared with other measuring points, indicating that these positions are barely affected by Phase A galloping. The waveforms of $A_{X11}$ and $A_{X14}$ at positions directly above and below the Phase A conductor have a phase difference of nearly 180°, and only the frequency component related to in-plane galloping (i.e., 0.5 Hz) is induced at these positions, which demonstrates that only in-plane galloping can be monitored at these specific positions. Both in-plane and out-of-plane galloping frequency components exist in the signals of $S_{2}$ and $S_{5}$, indicating that these positions can effectively monitor the galloping of all modes. Based on the above analysis, the following conclusions are drawn:

1. The magnetic field fluctuation near a certain phase conductor is mainly caused by the galloping of the conductor itself, and is barely affected by the galloping of other conductors. In other words, conductor galloping only affects measuring points within a short distance from the conductor, and has limited influence on measuring points far away.
2. The magnetic field fluctuation at positions directly above and below the conductor is mainly caused by in-plane galloping. When the measuring point deviates from the vertical alignment of the conductor, both in-plane and out-of-plane galloping jointly affect the magnetic field fluctuation at the measuring point.

Accordingly, a simplified expression of $A_{Xps}$ can be obtained for subsequent derivation and analysis:

(6) $$A_{Xps}\left(t\right)=\left\{\begin{array}{ll} A_{Xps}^{0}+n_{p}^{i}\sin\theta_{p}^{i}, & \mathrm{if}\;x_{s}=x_{p},\\ A_{Xps}^{0}+m_{p}^{i}\sin\theta_{p}^{i}+m_{p}^{o}\sin\theta_{p}^{o}, & \mathrm{if}\;|x_{s}-x_{p}|\leq l_{p}/3,\\ A_{Xps}^{0}, & \mathrm{if}\;|x_{s}-x_{p}|\geq 2l_{p}/3, \end{array}\right.$$

where $A_{Xps}^{0}$ denotes the magnetic field contribution of the stationary Phase *p* conductor to the measuring point *s*, $n_{p}^{i}$, $m_{p}^{i}$ are functions of the in-plane galloping amplitude while $m_{p}^{o}$ is a function of the out-of-plane galloping amplitude, and $\theta_{p}^{i}$, $\theta_{p}^{o}$ are related to the galloping frequency.

## 3. Galloping Monitoring Method by MF Distribution Characteristics

### 3.1. Anti-Current-Interference Sensor Array Layout

**Figure 4:**
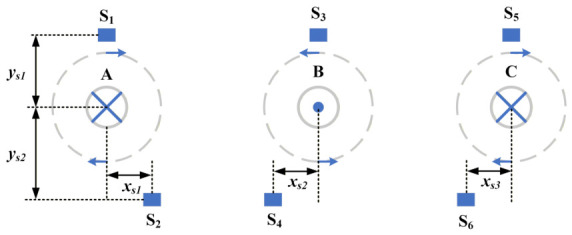
Layout of the proposed anti-current interference sensor array.

According to the conclusions drawn above, in ideal scenarios, only a sensor deployed at a position deviated from the vertical alignment near a conductor is required to monitor both the in-plane and out-of-plane galloping of the conductor. However, spatial magnetic field fluctuations are induced not only by conductor position changes but also by current variations in the conductor. When the current fluctuation in the transmission line presents periodic characteristics (e.g., low-frequency current oscillation with frequency close to the galloping frequency), the magnetic field fluctuation it causes at a certain spatial point is difficult to distinguish from that induced by conductor galloping. Therefore, under such conditions, an array structure is required to comprehensively determine the galloping state of the transmission line using magnetic field information from multiple measuring points. Combined with the conclusions obtained in Section 2.3, a magnetic field sensor array layout scheme is proposed: the magnetic field information captured by this array can effectively distinguish magnetic field fluctuations caused by current variations and conductor galloping. Taking the tower structure shown in Figure 1 as an example, the installation positions of the sensors are shown in Figure 4:

Specifically, the galloping state of each conductor is independently monitored by two sensors deployed above and below the conductor respectively. The upper sensor is installed on the cross-arm above the conductor insulator, maintaining a safe insulation distance from the conductor. On the premise of guaranteeing the safe insulation distance, the lower sensor is deployed at a position where the horizontal distance from the conductor is approximately 1/4 of the phase-to-phase distance. The magnetic field density expressions of these two sensors are given as follows:

(7) $$\begin{array}{cc} \; & B_{X_{1}}\left(t\right)=A_{X_{11}}\left(t\right)I_{1}\left(t\right)+A_{X_{21}}^{0}I_{2}\left(t\right)+A_{X_{31}}^{0}I_{3}\left(t\right),\\ \; & B_{X_{2}}\left(t\right)=A_{X_{12}}\left(t\right)I_{1}\left(t\right)+A_{X_{22}}^{0}I_{2}\left(t\right)+A_{X_{32}}^{0}I_{3}\left(t\right). \end{array}$$

When the sensors are deployed as shown in Figure 4, it can be obtained from the MF density expressions that $B_{X1}$ and $B_{X2}$ are instantaneous response quantities under homologous current excitation, and their time dimensions are fully aligned: the moment when the current changes is the moment when the two MF densities change synchronously, with no phase difference or time delay. Only the response amplitudes of the two quantities differ due to the difference in magnetic coupling coefficients at different positions. As indicated by (6), when the conductor gallops, the coefficients $A_{X11}$ and $A_{X12}$ present obvious time asynchrony: $A_{X11}$ only contains components related to in-plane galloping, while $A_{X12}$ contains both in-plane and out-of-plane galloping components, and the variations of $A_{X11}$ and $A_{X12}$ caused by in-plane galloping are not synchronized. Therefore, by comprehensively comparing the phase characteristics and frequency-domain characteristics of the MF density variations at the two positions, galloping detection can be realized while eliminating current interference.

### 3.2. Proposed Galloping Monitoring Method

As analyzed in Section 3.1, the magnetic field fluctuations caused by current variations show synchronous variation trends at the two positions shown in Figure 4, while the magnetic field fluctuations induced by conductor galloping present significant asynchrony. Accordingly, a galloping detection method immune to current fluctuation interference is proposed. First, (7) are rewritten taking the moment when $I_{1}$ reaches the maximum value as the reference time point:

(8) $$\begin{array}{cc} \; & B_{X_{1}}\left(t_{0}\right)=\left[A_{X_{11}}\left(t_{0}\right)+\alpha A_{X_{21}}^{0}+\beta A_{X_{31}}^{0}\right]I_{1}\left(t_{0}\right),\\ \; & B_{X_{2}}\left(t_{0}\right)=\left[A_{X_{12}}\left(t_{0}\right)+\alpha A_{X_{22}}^{0}+\beta A_{X_{32}}^{0}\right]I_{1}\left(t_{0}\right). \end{array}$$

Here, α and β represent the ratios of $I_{2}$ and $I_{3}$ to $I_{1}$ at this moment respectively, and the ratios are approximately −0.5 under three-phase balance conditions. In fact, the simulation results show that when $I_{1}$ reaches the maximum value, since $I_{2}$ and $I_{3}$ are far from sensors $S_{1}$ and $S_{2}$ with small current amplitudes, the values of $B_{Xs}\left(t_{0}\right)$ and $B_{X\max}$ are close (with an error less than 1%). Therefore, $B_{X\max}$ is commonly used to replace $B_{X}\left(t_{0}\right)$ in practical engineering applications. When a long time scale (covering multiple current cycles) is considered, (8) can be rewritten as

(9) $$\begin{array}{cc} \; & B_{{X_{1}}_{RMS}}\left(t\right)=\left[A_{X_{11}}\left(t\right)+\alpha A_{X_{21}}^{0}+\beta A_{X_{31}}^{0}\right]I_{1RMS}\left(t\right),\\ \; & B_{X_{2RMS}}\left(t\right)=\left[A_{X_{12}}\left(t\right)+\alpha A_{X_{22}}^{0}+\beta A_{X_{32}}^{0}\right]I_{1RMS}\left(t\right). \end{array}$$

Substituting the expression of $A_{Xps}$ and taking current fluctuations into consideration, the following equation can be obtained:

(10) $$\begin{array}{cc} \; & B_{{X_{1}}_{RMS}}\left(t\right)=\left({A^{0}}_{X_{11}}+{n_{1}}^{i}\sin{\theta_{1}}^{i}+C_{1}\right)\left(I_{1RMS}^{DC}+\Delta I_{1RMS}\right),\\ \; & B_{X_{2RMS}}\left(t\right)=\left({A^{0}}_{X_{12}}-{m_{2}}^{i}\sin{\theta_{1}}^{i}+{m_{2}}^{o}\sin{\theta_{2}}^{o}+C_{2}\right)\left(I_{1RMS}^{DC}+\Delta I_{1RMS}\right), \end{array}$$

where

(11) $$C_{1}=\alpha A_{X_{21}}^{0}+\beta A_{X_{31}}^{0},C_{2}=\alpha A_{X_{22}}^{0}+\beta A_{X_{32}}^{0}$$

Here $I_{1RMS}^{DC}$ represents the steady-state component of the current root mean square (RMS) value, and $\Delta I_{1RMS}$ denotes the fluctuating component of the current RMS value. Fourier transform is performed on (10), with F{·} defined as the Fourier transform operator, δ(f) as the unit impulse function, and $S_{\Delta I}\left(f\right)$ as the power spectral density of $\Delta I_{1RMS}\left(t\right)$. The in-plane galloping frequency is denoted as $f^{i}$ and the out-of-plane galloping frequency as $f^{o}$, and their respective frequency spectra are as follows:

(12) $$\begin{array}{cc} \; & B_{X_{1RMS}}\left(f\right)=\left(A_{X_{11}}^{0}+C_{1}\right)I_{1RMS}^{DC}\;\cdot \;\delta \left(f\right)+\left(A_{X_{11}}^{0}+C_{1}\right)\;\cdot \;S_{\Delta I}\left(f\right)+\\ \; & n_{1}^{i}I_{1RMS}^{DC}\;\cdot \;\frac{j}{2}\left[\delta \left(f+f^{i}\right)-\delta \left(f-f^{i}\right)\right]+n_{1}^{i}\;\cdot \;\left(S_{\Delta I}\left(f\right)*\frac{j}{2}\left[\delta \left(f+f^{i}\right)-\delta \left(f-f^{i}\right)\right]\right) \end{array}$$

(13) $$\begin{array}{cc} \; & B_{X_{2RMS}}\left(f\right)=\left(A_{X_{12}}^{0}+C_{2}\right)I_{1RMS}^{DC}\;\cdot \;\delta \left(f\right)+\left(A_{X_{12}}^{0}+C_{2}\right)\;\cdot \;S_{\Delta I}\left(f\right)\\ \; & -m_{2}^{i}I_{1RMS}^{DC}\;\cdot \;\frac{j}{2}\left[\delta \left(f+f^{i}\right)-\delta \left(f-f^{i}\right)\right]-m_{2}^{i}\;\cdot \;\left(S_{\Delta I}\left(f\right)*\frac{j}{2}\left[\delta \left(f+f^{i}\right)-\delta \left(f-f^{i}\right)\right]\right)\\ \; & +m_{2}^{o}I_{1RMS}^{DC}\;\cdot \;\frac{j}{2}\left[\delta \left(f+f^{o}\right)-\delta \left(f-f^{o}\right)\right]+m_{2}^{o}\;\cdot \;\left(S_{\Delta I}\left(f\right)*\frac{j}{2}\left[\delta \left(f+f^{o}\right)-\delta \left(f-f^{o}\right)\right]\right) \end{array}$$

Therefore, all possible frequency components in $B_{X_{1RMS}}$ and $B_{X_{2RMS}}$ can be extracted from (12) and (13), and the results are shown in Table 2:

It can be observed from the frequency domain characteristic analysis of $B_{X1RMS}$ and $B_{X2RMS}$ that frequency components associated with in-plane galloping exist in the spectra of both $B_{X1RMS}$ and $B_{X2RMS}$ but with different phases, while frequency components related to out-of-plane galloping only appear in $B_{X2RMS}$. Frequency components corresponding to random current fluctuations are present in both $B_{X1RMS}$ and $B_{X2RMS}$ with identical phases. The cross-term frequencies between in-plane/out-of-plane galloping and current fluctuations are not dominant frequency components due to their small amplitudes. Therefore, the occurrence of galloping can be comprehensively determined by combining spectrum and phase information.

### 3.3. Proposed Algorithm Procedure

**Figure 5:**
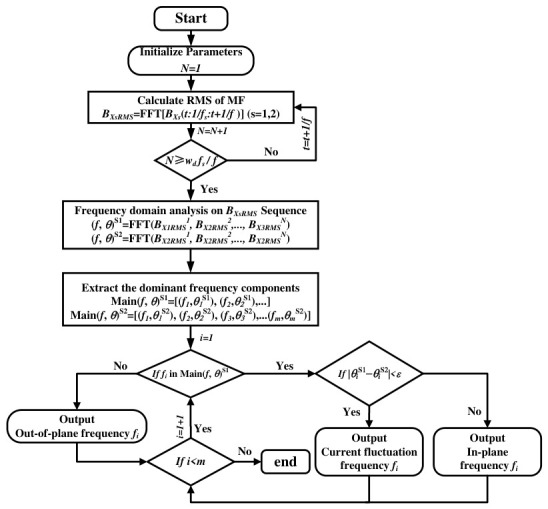
Flow chart of the proposed galloping detection algorithm.

Based on the above method, we design the algorithm flow chart for galloping detection as shown in Figure 5. We take the galloping detection of phase A conductor as an example for illustration, and the galloping detection algorithms for phase B and C are identical.

First, calculate the root mean square (RMS) value of the magnetic field output by the sensor per power cycle within the time window of length $w_{d}$ to form the MF density RMS sequence $B_{XsRMS}\left(N\right)$, where $f_{s}$ is the sampling frequency and *f* is the power frequency (50/60 Hz). Second, analyze the spectrum of the RMS sequences $B_{XsRMS}\left(N\right)$ output by the two sensors using Fast Fourier Transform (FFT) to obtain each frequency and its phase information. Then extract the respective dominant frequency components and their phase information. Traverse all dominant frequencies in the dominant frequency sequence $B_{X2RMS}$:

1. If a dominant frequency in $B_{X2RMS}$ does not exist in the dominant frequency sequence $B_{X1RMS}$, it is regarded as an out-of-plane galloping frequency and output.
2. If a dominant frequency in $B_{X2RMS}$ exists in $B_{X1RMS}$, judge whether its phase is consistent with the phase of the same frequency in $B_{X1RMS}$: if consistent, the frequency is judged to be related to current fluctuation; if inconsistent, it is detected as an in-plane galloping frequency and output. The algorithm ends after all dominant frequency components in $B_{X2RMS}$ are traversed.

## 4. Simulation Validation

### 4.1. Simulation Parameter Configuration for Real-World Scenarios

To verify the effectiveness of the proposed method, we conduct numerical simulations using actual transmission line parameters, which are listed in Table 3.

The magnetic field data are generated according to (4) and (5). To better replicate real-world scenarios, noise is added to the generated magnetic field data to keep the signal-to-noise ratio (SNR) of the magnetic field data at approximately 40 dB, which further validates the effectiveness of the proposed method in practical application scenarios. In this experiment, only the galloping of phase A conductor is taken as the analysis object, and the analysis for phase B and C conductors follows the same logic. In addition, in the simulation verification, the galloping frequencies of phase B and C conductors are set to be different from that of phase A, to verify the influence of galloping of phase B and C conductors on the magnetic field at the sensor near phase A.

### 4.2. Galloping Detection Under Low-Frequency Current Oscillation

**Figure 6:**
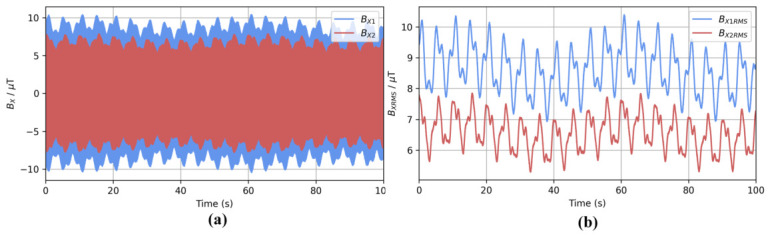
Fluctuation of magnetic field at sensors $S_{1}$ and $S_{2}$ under conductor galloping and low-frequency current oscillation: (**a**) fluctuation of MF intensity, (**b**) fluctuation of RMS value of MF intensity.

**Figure 7:**
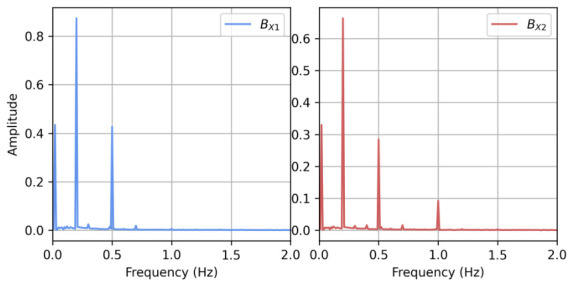
Frequency spectrum of RMS values of MF intensity at $S_{1}$ and $S_{2}$ under low-frequency current oscillation.

In practical scenarios, low-frequency oscillation may occur in the current due to weak grid damping, power fluctuation of large-scale grid-connected new energy sources, switching disturbance of high-power loads and other reasons, with the oscillation frequency ranging from 0.1 Hz to 2.5 Hz. The fluctuation of the generated spatial magnetic field also falls within this frequency band, so it is impossible to distinguish the magnetic field fluctuation caused by galloping from that caused by low-frequency current oscillation using a single sensor. Therefore, to verify the feasibility of the proposed magnetic sensor array-based galloping detection method under such scenarios, the current amplitude is set to oscillate at a low frequency around 500 A, with the oscillation frequency set to 0.2 Hz. On this basis, a 0.02 Hz current variation trend term is superimposed to make the overall current waveform more complex, which is closer to the actual operation characteristics of transmission lines. In this simulation scenario, the real-time variations of the MF density and the corresponding root mean square (RMS) values of MF density at sensors $S_{1}$ and $S_{2}$ are shown in Figure 6:

It can be seen from Figure 6 that the envelope of the MF density has a roughly similar shape to the root mean square (RMS) value of the MF density, and its fluctuation characteristics can reflect the low-frequency vibration caused by current and galloping. Fast Fourier Transform (FFT) is used to extract the spectrum of the RMS value of the MF density, and the obtained results are shown in Figure 7.

As can be observed from the spectrum, the main frequency components include 0.02 Hz, 0.2 Hz, 0.5 Hz and 1 Hz. At this point, current oscillation and galloping cannot be effectively distinguished solely from the spectrum. Therefore, we calculate the phase information of each frequency component at the two measurement points as shown in Table 4.

It can be seen from the table that the frequency components of 0.02 Hz and 0.2 Hz have identical phases in $B_{X1RMS}$ and $B_{X2RMS}$, indicating that these frequency components are caused by current oscillation; the phase of the 0.5 Hz component is inconsistent between $B_{X1RMS}$ and $B_{X2RMS}$, indicating that it corresponds to in-plane galloping; the 1 Hz frequency only exists in $B_{X2RMS}$, indicating that it corresponds to out-of-plane galloping. This result is consistent with the simulation settings, which verifies that the proposed method can effectively distinguish low-frequency current oscillation from conductor galloping. In addition, it can be seen from the spectrum that 0.4 Hz and 0.6 Hz components are not present in the main frequency components, indicating that the galloping of phase B and C conductors has negligible influence on the magnetic field at the sensors near phase A.

### 4.3. Galloping Detection Under Random Current Fluctuation

**Figure 8:**
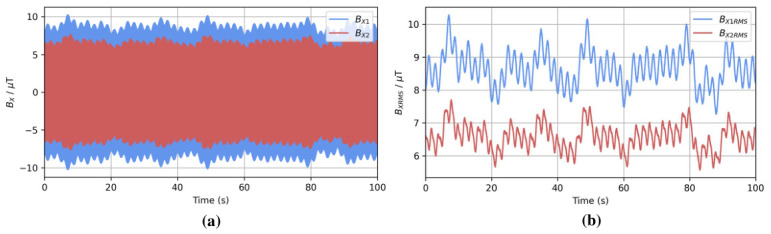
Fluctuation of magnetic field at sensors $S_{1}$ and $S_{2}$ under conductor galloping and random current fluctuation: (**a**) fluctuation of MF intensity, (**b**) fluctuation of RMS value of MF intensity.

**Figure 9:**
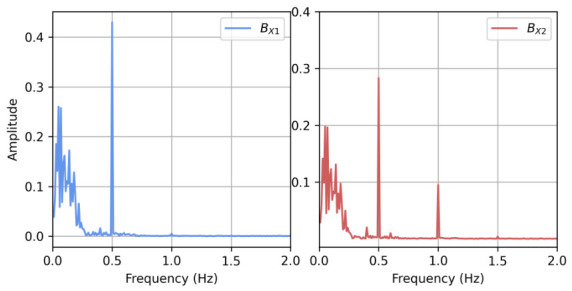
Frequency Spectrum of RMS Values of MF Intensity at $S_{1}$ and $S_{2}$.

In practice, under actual operating conditions, conductor galloping lasts for a long time, during which the current fluctuation presents certain randomness. Therefore, to further verify the feasibility of the proposed method under complex current operating conditions, we designed a simulation experiment under random current fluctuation. The real-time MF density, the root mean square (RMS) value of MF density and the corresponding spectrum at sensors $S_{1}$ and $S_{2}$ are shown in Figure 8 and Figure 9 respectively.

It can be seen that due to the random fluctuation of the current, a large number of frequency components appear in the low-frequency part (below 0.25 Hz) of the spectrum. We extract the dominant frequency components in the range of 0.1 Hz to 3 Hz and their corresponding phase information, and the obtained results are shown in Table 5:

It can be seen from the table that the phase of frequency components related to current fluctuation is consistent in $B_{X1RMS}$ and $B_{X2RMS}$, while there is a significant phase difference between $B_{X1RMS}$ and $B_{X2RMS}$ for frequencies related to in-plane galloping, and frequencies related to out-of-plane galloping only appear in $B_{X2RMS}$. This conclusion further verifies the galloping monitoring capability of the proposed method under strong current fluctuation interference.

### 4.4. Performance Evaluation of the Proposed Method

To comprehensively evaluate the applicability of the proposed method under various scenarios, we generate 100 independent samples for simulation validation. These samples cover three categories: (1) current fluctuation without galloping (20 samples), where only current fluctuation is present and the conductor remains stationary, serving to evaluate the false alarm rate; (2) galloping without current fluctuation (20 samples), consisting of 10 single-conductor galloping cases and 10 multi-conductor simultaneous galloping cases; and (3) current fluctuation with galloping (50 samples), comprising 30 single-conductor cases and 20 multi-conductor cases. It should be noted that the galloping amplitudes cover a wide range from mild (in-plane 2 m, out-of-plane 1 m) to extreme (in-plane 10 m, out-of-plane 5 m). In the multi-conductor cases, the amplitude and phase of each phase are set independently and randomly. Moreover, a 20 s sliding window was adopted for FFT analysis (long enough to capture galloping frequencies as low as 0.1 Hz), resulting in a detection delay equal to the window length of 20 s. Based on the phase consistency of the current-modulation components at the two sensors (as shown in Table 2), a phase difference threshold of ±50° was selected through scanning to separate galloping signals from current fluctuations. This relatively large threshold was chosen to accommodate the slight increase in phase difference caused by sensor installation errors and thereby prevent false alarms, while it remains well below 180° because the phase difference between the upper and lower sensors for in-plane galloping is intrinsically 180°.

The detection results are summarized in Table 6. As shown, no false alarm occurred in the 20 current-fluctuation-only samples (false alarm rate = 0%). Among the 100 normal samples, galloping was successfully detected in 97 cases, yielding an overall detection accuracy of 97%. The three missed detections all occurred under conditions where the in-plane amplitude was near the minimum detectable boundary (approximately 2 m in-plane and 1 m out-of-plane under the experimental conditions of this study) combined with large current fluctuations, indicating that the detection difficulty increases when the galloping amplitude approaches the detection limit. The above results further confirm the accuracy and feasibility of the proposed method for galloping detection.

## 5. Experimental Validation

### 5.1. Experimental Setup

**Figure 10:**
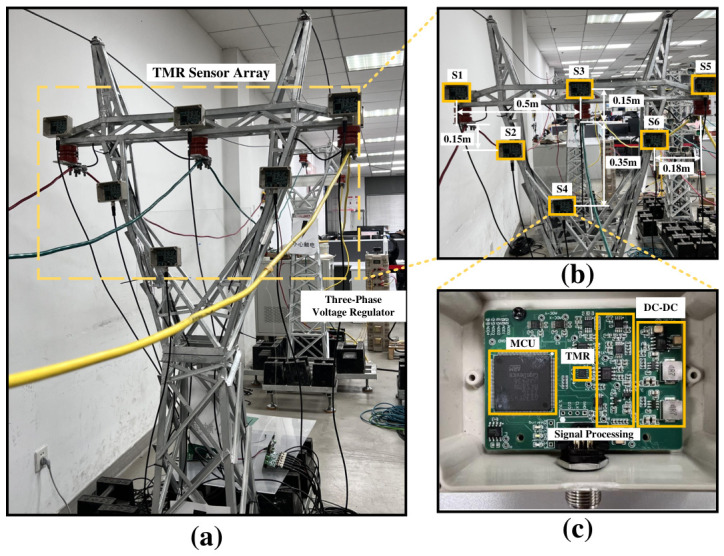
Construction of the experimental system: (**a**) scaled-down model in laboratory environment, (**b**) placement of the sensor array, and (**c**) hardware composition of the magnetic field sensor.

To further verify the effectiveness of the proposed method, we built an equal-proportion scaled-down model of an actual transmission tower (a section of 110 kV transmission line) as shown in Figure 10 in the laboratory environment, with a scaling ratio of approximately 20:1. The scaling was not based on strict dynamic similarity, but on the principle of magnetic field equivalence. According to the Biot–Savart law, the magnetic flux density *B* around a long straight conductor is proportional to the ratio of the conductor current *I* to the sensor distance *r*. Therefore, by maintaining the same I/r as in the field, the laboratory magnetic environment can be made comparable to that of a real transmission line. For a typical 110 kV line with a normal operating current around 200 A and a planned sensor distance of about 2 m from the conductor suspension point, the magnetic field is approximately 20 μT. In the 20:1 scaled-down model, the sensor was placed 0.15 m from the conductor (about 1/20 of 2 m) and the current was controlled at 15 A, yielding the same I/r and a magnetic field of approximately 20 μT. The background noise measured under no-load conditions was about 0.05 μT, which falls within the 0.05–0.1 μT range reported for actual transmission lines [28], thus ensuring a comparable signal-to-noise ratio. The conductor motion amplitude was also scaled down geometrically by a factor of 20: real-world galloping amplitudes typically range from 2 to 5 m, corresponding to 0.1–0.25 m in the experiment, which maintains equivalence in spatial displacement.

The material of the scaled tower is consistent with that of the actual tower, with the same magnetic permeability, which can well simulate real-world scenarios. A three-phase voltage regulator is used to adjust the voltage to change the current in the line, and the load consists of six resistors with a resistance of 5 Ω and a maximum power capacity of 4 kW connected in series. Six magnetic sensing units are placed at the positions shown in Figure 10b. Considering the installation limitation at the restricted positions of the tower, we adjusted the position of the sensors: if placed according to the layout in Figure 4, there is no support at the position of sensor $S_{4}$, so it is adjusted downwards to a position with support. It still remains at the position slightly to the left below the phase B conductor, where the galloping of phase B can still be detected without interference from phase A and C conductors, which does not conflict with the previous theoretical design. Each magnetic sensing unit consists of a TMR2103 tunnel magnetoresistance (TMR) sensor and a signal processing circuit. The signal processing circuit includes an instrumentation amplifier, a Butterworth band-pass filter, and an analog-to-digital conversion (ADC) circuit. The TMR2103 sensor outputs a differential voltage signal, so the instrumentation amplifier is used to filter out the common-mode interference from the environment to the differential signal. The center frequency band of the Butterworth band-pass filter is set to 50 Hz with a bandwidth of 5 Hz, which is used to retain the power frequency signal while filtering out low-frequency environmental noise (such as geomagnetic field) and high-frequency noise (such as radio interference) in space. The analog-to-digital conversion module is processed by a Microcontroller Unit (MCU), and the root mean square (RMS) value of the magnetic flux density used in the algorithm mentioned above is also calculated by the MCU.

### 5.2. Experiments

**Figure 11:**
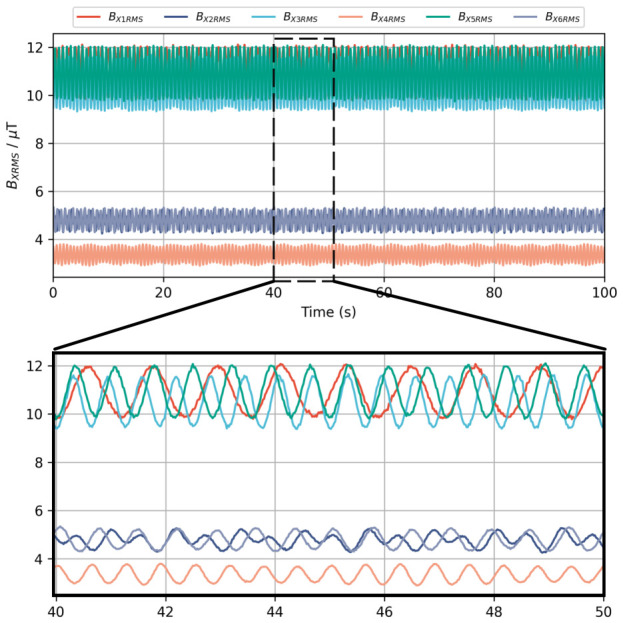
Time-domain fluctuation of RMS values of MF intensity of six magnetic field sensors under galloping.

**Figure 12:**
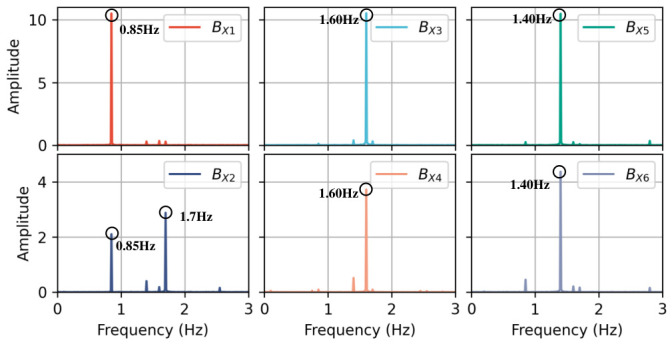
Frequency spectrum of RMS values of MF intensity at six sensors.

We first verified the galloping detection performance of the proposed method under no current fluctuation. In real scenarios, the non-uniform ice coating on three-phase conductors and differences in line parameters (such as sag, etc.) will lead to certain differences in the modes and frequencies of three-phase galloping. To better simulate the galloping conditions in real scenarios, we set the galloping mode of phase A conductor as the second-order mode, with the frequencies of in-plane galloping and out-of-plane galloping being 0.85 Hz and 1.7 Hz, respectively, which is a typical 1:2 internal resonance galloping mode. The in-plane and out-of-plane galloping frequencies of phase B are both set to 1.6 Hz, and the galloping frequency of phase C is set to 1.4 Hz. The three-phase current remains constant, and the measurement results are shown in Figure 11:

Figure 11 shows the real-time variation of the RMS value of MF density of the six sensors. It can be seen that due to the conductor galloping, the RMS value of MF density at the sensor presents sinusoidal fluctuation characteristics related to galloping. The values of sensors $S_{2}$, $S_{4}$ and $S_{6}$ located below the conductors are smaller than those of $S_{1}$, $S_{3}$ and $S_{5}$ located above the conductors because they are farther away from the conductors. Spectrum analysis is performed on the RMS value of the output magnetic field of the sensors, and the obtained results are shown in Figure 12.

It can be seen that frequencies related to the galloping of phase A conductor appear in the spectra of $S_{1}$ and $S_{2}$, and the out-of-plane galloping frequency only exists in $S_{2}$, which is consistent with the law found in the expression of (6). Similarly, components related to the galloping frequency of phase B are detected in $S_{3}$ and $S_{4}$, and components related to the galloping frequency of phase C are detected in $S_{5}$ and $S_{6}$. It is worth noting that no galloping frequencies of non-target phases appear in the main spectrum of each corresponding sensor, which further verifies the conclusion in Section 2.3. The above results prove the feasibility of the proposed method and the sensor array layout in galloping detection.

**Figure 13:**
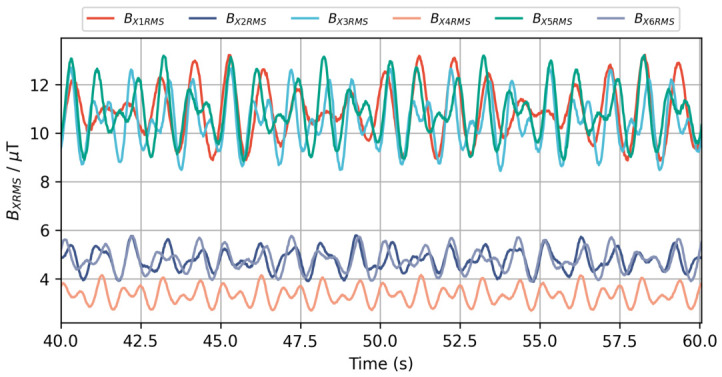
Time-domain fluctuation of RMS values of MF intensity of six magnetic field sensors under conductor galloping and low-frequency current oscillation.

**Figure 14:**
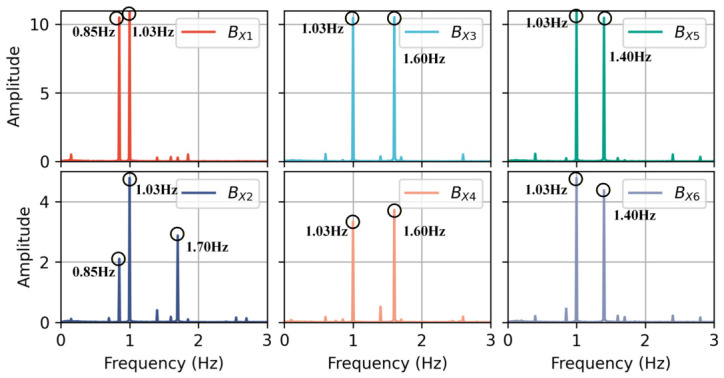
Frequency spectrum of RMS values of MF intensity at six sensors under current fluctuation.

To further verify the applicability of the proposed method in more complex scenarios, we adjust the three-phase output current through a three-phase voltage regulator, with a current variation frequency of approximately 1 Hz. Meanwhile, we set the galloping of the three-phase conductors according to the galloping parameters mentioned above, to observe the galloping detection performance under current fluctuation. Figure 13 shows the variation of the RMS value of MF density of the 6 sensors within 40–60 s under this condition. Compared with Figure 11, it can be seen that the magnetic flux density waveform in this case is superimposed with more complex frequency components, resulting in waveform distortion. As can be seen from the respective spectrum analysis (shown in Figure 14), a frequency component of 1.03 Hz is superimposed in the output magnetic field of each sensor. The phase information of all frequency components is calculated, and the obtained results are shown in Table 7.

It can be seen from Table 7 that the phase of the 1.03 Hz frequency component is consistent in $S_{1}$ and $S_{2}$, $S_{3}$ and $S_{4}$, $S_{5}$ and $S_{6}$. According to the above analysis, this frequency component is caused by current fluctuation and is not a galloping-related frequency. After removing this frequency, the remaining frequency components are consistent with the galloping frequency parameters we set, which proves the effectiveness of the proposed method for galloping detection under current fluctuation.

To further evaluate the applicability of the proposed method under different scenarios, the experimental validation was designed following a similar structure as the simulation. A total of 50 experimental runs were conducted on the 20:1 scaled-down platform, comprising cases with current fluctuation only and no galloping, cases with galloping only and no current fluctuation, and cases with both current fluctuation and galloping. The results show that galloping events were correctly identified in 48 out of the 50 runs, yielding a detection accuracy of 96%, with no false alarms observed in the pure current-fluctuation cases. The two missed detections occurred under conditions where the magnetic field variation induced by galloping was close to the minimum detectable level of the measurement system, which corresponds to a galloping amplitude of approximately 0.02 m in the laboratory setup. These experimental findings further confirm the potential of the proposed method for practical galloping monitoring.

## 6. Uncertainty Analysis in Engineering Scenarios

The feasibility of the galloping detection method proposed in this paper has been verified through numerical simulations and laboratory experiments. However, real-world scenarios are often more complex; therefore, the uncertainties that may arise in practical field environments need to be thoroughly discussed. Torsional motion is disregarded in this study because the associated spatial displacement is small and difficult to detect with magnetic sensors. Moreover, field experience consistently shows that large-amplitude transverse oscillation is the primary hazard for transmission lines, while torsion appears only as a secondary coupled effect. In future work, combining magnetic sensing with additional measurements (e.g., tilt sensors) could be explored to capture the torsional component. A single-conductor model is used instead of a bundle-conductor model. This simplification is justified by the far-field measurement geometry: when the sensors are mounted on the tower at a distance substantially larger than the sub-conductor spacing, the resultant magnetic field of the bundle can be approximated by that of a single conductor with an error below 1%. The relative motion among sub-conductors and its subtle influence on the magnetic field lie beyond the scope of this proof-of-concept study and are left for future investigation. As for environmental factors, ice shape and wind direction are not the focus of this paper because the objective is to detect galloping once it has occurred, rather than to study the aerodynamic excitation mechanism that triggers it; moreover, the magnetic measurement principle adopted here does not itself depend on these specific meteorological details. In practical field environments, the diversity of current operating conditions can pose challenges to magnetic-field-based galloping detection methods. However, the core assumption of this paper—that current-induced magnetic field variations share the same phase at the upper and lower sensors of the same phase conductor—has been verified through simulations under various non-ideal current scenarios, including three-phase imbalance, harmonic injection, fault transients, and interference from adjacent parallel lines. The results confirm the robustness of this assumption and demonstrate the good adaptability of the proposed galloping detection method under different current operating conditions.

The proposed sensor layout strategy remains adaptable across different span lengths, tower types, phase spacings, and galloping orders. Shorter spans slightly increase adjacent-phase interference, so the lower sensor should be placed closer to the point directly below the target phase to suppress this effect. The strategy also applies to various tower configurations: for triangular arrangements the same one-above–one-below placement is maintained, while for double-circuit towers lacking direct cross-arm support the sensors can be mounted on the tower body. Reduced phase spacing similarly increases adjacent-phase interference, again requiring the lower sensor to be positioned nearer the target phase. Galloping order mainly influences the vertical sensor distance: high-order modes produce smaller displacements and thus weaker magnetic signatures, so the sensor should be installed closer to the suspension point when historical data indicate frequent high-order events. Installation errors are not a significant concern. Simulations with ±5% distance and ±5° angular errors increase the phase differences between the two sensors above and below the same conductor for the current-modulation and galloping components by at most 3° and 5°, respectively, both far below the typical 50° discrimination threshold. Any residual offset can be eliminated through initial calibration with a known current. Regarding long-term stability and temperature, the TMR2103 sensor features a sensitivity temperature coefficient of approximately −13 ppm/°C and long-term drift better than ±5% full scale. The combined drift caused by long-term monitoring and temperature variations is on the order of nanotesla (nT), making the drift over the 20 μT measurement range negligible compared with the minimum detectable galloping signal. Thermal–structural simulation indicates that sensor position drift due to ambient temperature is less than 2 mm (equivalent to <0.01 μT field variation). For electromagnetic interference, a band-pass filter (center 50 Hz, bandwidth 10 Hz) suppresses geomagnetic and radio-frequency noise, yielding a signal-to-noise ratio above 40 dB.

## 7. Conclusions

Accurate and reliable online monitoring of transmission line galloping is the core requirement to ensure the safe and stable operation of large-scale power grids. Aiming at the two core bottlenecks of existing galloping monitoring technologies based on magnetic field sensing, i.e., no theoretical support for sensor deployment and high vulnerability to false detection caused by current fluctuation interference, this paper proposes a non-contact transmission line galloping monitoring method based on spatial magnetic field distribution characteristics, which provides a feasible technical solution for transmission line galloping monitoring under complex operating conditions. Firstly, this paper establishes a coupling mapping model between conductor galloping and the surrounding spatial power frequency magnetic field distribution, and quantitatively analyzes the sensitivity difference of different spatial points to two typical galloping modes of conductors: in-plane and out-of-plane galloping. On this basis, an optimal layout strategy of the magnetic sensing array is proposed, which provides a theoretical basis for the engineering deployment of magnetic field sensors at the transmission tower side, and solves the pain point that the existing technology lacks layout guidance for practical implementation. Secondly, aiming at the problem of inaccurate galloping discrimination caused by magnetic field distortion under operating conditions such as random current fluctuation and fault transient, this paper compares and analyzes the magnetic field frequency domain and phase distribution characteristics under two scenarios: current fluctuation and normal galloping, and proposes a galloping detection algorithm integrating multi-node magnetic field frequency domain features and phase difference information. Utilizing the core feature that the magnetic field change caused by current fluctuation has global phase synchronization in space, while the magnetic field change caused by galloping has obvious phase difference among multi-sensor nodes, the accurate identification of galloping signals under current interference is realized, which fundamentally eliminates the risk of false detection caused by current fluctuation.

To verify the effectiveness of the proposed method, this paper first carries out multi-scenario simulation verification based on the engineering parameters of actual 500 kV transmission lines. The simulation results show that the proposed method can effectively identify galloping and its frequency characteristics under typical interference conditions such as low-frequency current oscillation and random fluctuation, which verifies the feasibility of the method under real power grid parameters. Secondly, a laboratory verification platform with equal scale reduction of actual transmission towers is built, and galloping simulation experiments under two scenarios of steady-state current and current fluctuation are carried out using self-developed magnetic field sensing elements. The detection results of galloping frequency and mode obtained from the experiments are consistent with the preset parameters, which further verifies the reliability of the proposed method in practical application.

## Figures and Tables

**Table 1:** Comparison of different monitoring methods for conductor galloping.

Comparison Dimension	Magnetic Field Sensing	IMU	FBG	Vision-Based
Installation	Non-contact, fixed on tower	Contact, mounted on conductor	Contact, fiber bonded on conductor	Non-contact, camera on tower or ground
Cost	Low (low-cost sensors and acquisition)	Medium (sensors, power, wireless)	High (expensive interrogator)	Medium (cameras and processing)
Environmental adaptability	All-weather, unaffected by light, rain, fog, ice	Affected by severe vibration, thermal drift	Needs temperature compensation; fiber fragile under icing	Severely affected by light, fog, rain, snow, night
Maintenance difficulty	Low (fixed, long maintenance-free interval)	High (battery replacement, communication)	Medium (fiber fragility)	Medium (lens cleaning, lighting maintenance)
Damage risk under galloping	Very low (non-contact, away from conductor motion)	High (sensor moves violently, prone to detachment/damage)	Medium–high (fiber bends with galloping, prone to break under icing)	Low (non-contact, but lens may be ice-covered or mechanically shocked)

**Table 2:** Frequency components and their characteristics in $B_{X1\mathrm{RMS}}$ and $B_{X2\mathrm{RMS}}$.

Frequency	Source	Presence in $B_{X1\mathrm{RMS}}$ and $B_{X2\mathrm{RMS}}$
f=0	DC offset	Yes
$f=\pm f_{1}^{c},\pm f_{2}^{c},\ldots$	Random current fluctuation	Yes, same phase
$f=\pm f^{i}$	In-plane galloping	Yes, different phases
$f=\pm f^{o}$	Out-of-plane galloping	Only present in $B_{X2RMS}$
$f=f_{1}^{c}\pm f^{i},\;f_{2}^{c}\pm f^{i},\ldots$	Cross term between in-plane galloping and current fluctuation	Yes, small amplitude, non-dominant frequency
$f=f_{1}^{c}\pm f^{o},\;f_{2}^{c}\pm f^{o},\ldots$	Cross term between out-of-plane galloping and current fluctuation	Only present in $B_{X2RMS}$, small amplitude, non-dominant frequency

**Table 3:** Parameter settings in simulations.

Parameter	Value
Voltage Level (*U*)	500 kV
Current Range ($I_{p}$)	500 A
Span Length (*L*)	400 m
Phase Spacing ($l_{p}$)	12.6 m
Tower Height ($y_{t}$)	45 m
Vertical distance between sensors and conductors ($y_{s}$)	5 m
Horizontal distance between sensors and conductors ($x_{s}$)	3 m
In-plane Galloping Amplitude ($A_{i}$)	6 m
Out-of-plane Galloping Amplitude ($A_{o}$)	3 m
Phase Difference between In-plane and Out-of-plane Galloping (Δφ)	60°
Frequency of In-plane Galloping ($f_{1}^{i}$)	0.5 Hz
Frequency of Out-of-plane Galloping ($f_{1}^{o}$)	1 Hz
Galloping Frequency of Phase B	0.4 Hz
Galloping Frequency of Phase C	0.6 Hz

**Table 4:** Phase Comparison of dominant frequency components.

Frequency Component	Phase of $B_{X1\mathrm{RMS}}$ (°)	Phase of $B_{X2\mathrm{RMS}}$ (°)
0.02 Hz	−89.04	−89.07
0.2 Hz	−44.74	−44.84
0.5 Hz	−179.40	0.12
1 Hz	\	119.46

**Table 5:** Phase Comparison of Dominant Frequency Components.

Frequency Component	Phase of $B_{X1\mathrm{RMS}}$ (°)	Phase of $B_{X2\mathrm{RMS}}$ (°)
0.1 Hz	100.19	100.04
0.14 Hz	26.53	26.68
0.18 Hz	−120.11	−120.16
0.5 Hz	179.82	0.98
1 Hz	\	119.02

**Table 6:** Detection performance of the proposed method.

Performance Indicator	Value	Remark
Detection accuracy	97% (97/100)	Correct judgments of galloping presence/absence
False alarm rate	0% (0/100)	False alarm when no galloping present
Missed detection rate	3% (3/100)	Galloping present but not detected
Minimum detectable galloping amplitude	In-plane 0.5 m, out-of-plane 1 m	Under SNR ≥ 30 dB and missed detection rate ≤ 5%

**Table 7:** Frequency and phase results of each sensor under current fluctuation.

Phase A		Phase B		Phase C	
$S_{1}$ (f, θ)	$S_{2}$ (f, θ)	$S_{3}$ (f, θ)	$S_{4}$ (f, θ)	$S_{5}$ (f, θ)	$S_{6}$ (f, θ)
(0.85, −179.95)	(0.85, 3.82)	(1.03, −90.37)	(1.03, −89.92)	(1.03, −89.47)	(1.03, −89.44)
(1.03, −89.95)	(1.03, −89.68)	(1.60, 179.28)	(1.60, −18.62)	(1.40, −179.12)	(1.40, −33.74)
\	(1.70, 121.33)	\	\	\	\

Note: *f* is in Hz, θ is in °, \ indicates no corresponding frequency component.

## Data Availability

Data is unavailable due to privacy or ethical restrictions.
